# Prevention of Mother-to-Child HIV Transmission Internationally^1^

**DOI:** 10.3201/eid1011.040623_01

**Published:** 2004-11

**Authors:** Nathan Shaffer, Michelle McConnell, Omotayo Bolu, Dorothy Mbori-Ngacha, Tracy Creek, Ralph Ntumy, Loeto Mazhani

**Affiliations:** * Centers for Disease Control and Prevention, Atlanta, Georgia, USA;; † Centers for Disease Control Kenya, Nairobi, Kenya;; ‡ Botswana Ministry of Health National PMTCT Program, Gaborone, Botswana;; § Nyangabgwe Hospital, Francistown, Botswana

**Keywords:** infectious diseases, women, conference summary

Data from the Joint United Nations Programme on HIV/AIDS (UNAIDS) indicate that in 2003, 34–46 million people were living with HIV infection, and three fourths of these cases were in sub-Saharan Africa. Approximately 2.1–2.9 million children were living with HIV/AIDS. HIV transmission in sub-Saharan Africa is predominately heterosexual, and by the end of 2002, women represented 58% of HIV cases. UNAIDS estimates that in many African countries <1% of pregnant women receive needed antiretroviral prophylaxis to prevent mother-to-child HIV transmission (PMTCT). This has a substantial impact on the death rate in children, with previous gains reversed for children <5 years of age in several countries.

Without intervention, the risk of mother-to-child HIV transmission is 30%–35%. With antenatal HIV testing, combination antiretroviral drugs, and safer infant feeding, the risk can be reduced to 1%–2%. Simplified, short-course interventions can reduce PMTCT transmissions to 15%–20%. Interventions for PMTCT should also be provided in the broader context of prevention, including primary prevention of HIV, preventing unintended pregnancies, and care and support to HIV-infected women and their families.

## U.S. Government Response to Global Mother-to-Child HIV Transmission

In 2002, President George W. Bush introduced the International Mother and Child HIV Prevention Initiative. This initiative was coordinated across several U.S. government agencies including the Centers for Disease Control and Prevention (CDC) and U.S. Agency for International Development. The initiative focused on 14 countries in Africa and the Caribbean with high rates of HIV/AIDS. The goals of the initiative were to reduce mother-to-child transmission by up to 40%; support expanding national PMTCT programs; support linking PMTCT services with antiretroviral treatment and care for mothers, infants, and family members (“PMTCT-plus”); and reach up to 1 million women annually.

Core interventions include routinely recommending HIV counseling and testing at antenatal clinics, short-course antiretroviral prophylaxis for HIV-positive mother-infant pairs, counseling and support for safe infant feeding practices, and counseling for family planning. Additional interventions include prevention strategies for HIV-negative pregnant women and community mobilization to increase uptake and decrease stigma. By 2003, all 14 countries had started to provide services, and this initiative is now a major activity under the more comprehensive President's Emergency Plan for AIDS Relief, which targets the same 14 countries plus Vietnam.

## Implementing PMTCT Programs Internationally

### Case Study in Kenya

Kenya has a population of 31.1 million, with 1.2 million births every year. Of the 2.2 million people living with HIV/AIDS in Kenya, 1.4 million are women. The most rapidly growing population becoming infected with HIV is women. HIV-positive women give birth to 118,000 children annually. An estimated 35,000–40,000 of those infants are HIV-positive. Ten percent of reported HIV/AIDS cases in Kenya are in children <5 years of age. PMTCT interventions include antiretroviral drug prophylaxis, optimal obstetric care, infant feeding counseling, and family planning. Replacement feeding (as opposed to breastfeeding) is only recommended in environments where it is acceptable, feasible, sustainable, and safe. Through the CDC Global AIDS Program in Kenya, 18,000 antenatal women have learned their HIV status, and 50% of those who are HIV-positive have received prophylactic antiretroviral drugs. Barriers to testing include a lack of spousal support, fear of partner violence, and fear of disclosure and the stigma that may accompany it.

### Case Study in Botswana

Botswana's 2003 surveillance data show that 37.4% of women attending antenatal clinics are HIV-positive. Botswana has had a national PMTCT program since 2001 and an expanding antiretroviral treatment program since 2002. Both programs are free to patients. All pregnant women can receive HIV counseling and testing. Antiretroviral prophylaxis for women and infants and infant formula are provided for HIV-positive women. Although 95% of pregnant women attend antenatal clinics and deliver in health facilities, uptake of PMTCT has been low. A CDC-Botswana survey of pregnant women was performed to explore factors influencing HIV test acceptance. Factors predicting acceptance included higher educational level, attendance at urban clinics, greater knowledge about PMTCT, planned pregnancy, discussing HIV testing with others, and knowing others who had received PMTCT or antiretroviral therapy.

These presentations highlight the successes of PMTCT programs as well as continuing challenges. There continues to be a need for program evaluation, operational research, and expanded PMTCT services in order to maximally prevent mother-to-child HIV transmission.

